# Dermatological Needs in an Urban Free Health Care Setting

**DOI:** 10.7759/cureus.31203

**Published:** 2022-11-07

**Authors:** Jason Patel, Thomas Kozar, Josaih Sowell, Mary E Chambers, Om Patel, Tiffany Mayo

**Affiliations:** 1 Dermatology, University of Alabama at Birmingham Heersink School of Medicine, Birmingham, USA; 2 Department of Dermatology, University of Alabama at Birmingham, Birmingham, USA

**Keywords:** healthcare disparities, urban health services, medically uninsured, health services accessibility, dermatology

## Abstract

Introduction

A large proportion of the United States' underinsured population relies on free health clinics for their health care needs. With only a few free health clinics nationwide hosting specialty clinics, a small subset of which are dermatology clinics, there is a dearth of information in the literature on which dermatological pathologies and treatment modalities are most common in this setting. The purpose of this study was to establish the most common dermatological conditions and treatments in the free health care setting as well as understand which facets of care need improvement.

Methods

A total of 57 patients with dermatological findings were identified at an urban student-run free health clinic in the southern United States in the past two years (2019-2021). Information reviewed for each patient included general demographics, chief complaint, medical/surgical history, treatments/procedures required for each visit, treatments/procedures available for each visit, referrals, and follow-up rate. Qualitative analysis was performed.

Results

The median age of the patients that presented with dermatological findings was 40 while the most common ethnicities were white (26.2%), Hispanic/Latino (28.6%), and black (28.6%). The most common chief complaints were rashes and cysts with a majority (63.2%) of these patients presenting to this particular clinic for the first time. Seven patients (12.3%) were unable to receive treatment due to expense, procedure unavailability, or an unknown reason. The most common treatment prescribed included a topical steroid. A majority (71.9%) of the patients were unable to follow up as scheduled. A majority of patients (81.2%) that were able to follow up were adherent to their prescribed medication.

Conclusion

Although dermatological conditions are plentiful in the free health care setting, the literature currently contains no information regarding this topic. This may be due to low patient follow-up rates and inadequately charted outcomes on often outdated electronic health records. In order to best care for dermatology patients in this setting, it is necessary to understand the barriers to care and available treatment options.

## Introduction

Free health clinics are a valuable source of health care as well as a necessary safety net for many patients. Functioning as 501(c)3 nonprofit organizations, these clinics provide primary care services, mental health resources, pharmaceutical care, and dental care to populations that would otherwise be forced to go without [1,2].

With a growing prevalence of nonprofit clinics in the United States, this model of healthcare delivery is a vital component of the national effort to improve medical access for patients in need. Further growth of free clinics is especially important for those who may find it difficult to receive care due to lack of transportation, change in insurance status, or prohibitive cost. A 2017 report from the National Hospital Ambulatory Medical Care Survey (NAMCS) further emphasizes the need for specialists in free clinics, stating that an estimated 10% of all primary care visits result in a specialist referral [3]. While many free clinics have partnered specialists willing to take cases, patients cannot always pay copays or find transportation to these secondary visits. Thus, in addition to providing necessary routine and preventative medical care it is important that free clinics offer extended access to other areas of medical care via on-site specialty-specific clinic days run by volunteer physicians.

The literature currently contains limited studies about free health clinics, with no studies surrounding the practice of dermatology within these clinics. Dermatology concerns, however, are very commonplace in the primary care setting with up to a third of complaints being dermatological in nature [4,5]. It is pertinent that such studies are done to better address the dermatological needs of underserved patients. This will also help ensure that volunteer dermatologists, primary care physicians, and clinic staff are well-equipped to provide appropriate dermatological care and services when necessary. The goal of this study was to not only recognize the various dermatological presentations in this underserved population, but also to evaluate the effectiveness of the care provided at the free health clinic by assessing disease resolution at follow-up.

## Materials and methods

Patients were selected from an urban, student-run free health clinic, connected to an academic medical center in the southeastern United States during regular clinic days (primary care physicians) and/or during dermatology specialty clinic days (board-certified dermatologists). Out of 1738 total patient visits between January 1st, 2019, and December 31st, 2021, 71 (4.1%) patients with dermatology complaints were identified. Dermatology complaints included any complaint with key words describing skin, hair, nails, or cosmetics [6]. Of those 71 patients, 57 patients had sufficient information in their health records to be analyzed. Information that was determined to be sufficient included a minimum of patient complaint, diagnosis, and treatment. There were 61 patient encounters involving these 57 patients, with four of these patients presenting twice.

Information reviewed for each patient encounter included four categories with the following criteria as shown in Table 1: demographics, history of present illness, past medical history, and treatment/follow-up. Patients self-identified as male, female, or other, and also indicated their ethnicity as White, Hispanic/Latino, Black, Asian, or other/multiracial. Marital status was documented as single, married, or divorced, and level of education was documented as less than high school, high school/General Educational Development credential (GED), or college. Distance of residence or shelter for each patient was calculated in miles based on reported zip code. Data were also collected on a patient’s insurance status and number of previous visits to the free clinic. For each patient, the location of the pathology on the body was determined to be on the head/face, upper extremities, anterior torso, posterior torso, lower extremities, or anogenital area. Any past dermatological disease was noted and included any documented dermatological condition for which the patient was not presenting. This was also true for past hospitalizations and surgeries. Additionally, comorbidities were noted, including any psychiatric history and smoking status. Procedures performed and treatments received only included treatments performed for the presenting dermatology complaint. Follow-up included expected follow-up date and the actual date patients followed up. Referrals were classified as referrals to other physicians or organizations.

**Table 1:** Content Analyzed

Demographics	History of Present Illness	Past Medical History	Treatment/Follow-up
Gender	Year Seen in Clinic	Dermatological Disease	Procedures performed
Ethnicity	Chief Complaint	Hospitalizations	Treatments Received
Marital status	Clinic Data	Surgeries	Follow-up
Level of Education	Location of Pathology	Comorbidities	Referrals
Distance of residence		Allergies	

Each chief complaint and past dermatology disease was classified as dermatitis, growth/cysts, acne, concerns for neoplasm, infections, or alopecia/hyperpigmentation. The classification of dermatitis included any general skin irritation, itchiness, or rash that did not fit the criteria for other classifications. A growth or cyst was defined as any raised or fluid-filled lesion that did not fit the criteria for other classifications. The classification of infection included skin conditions that were a direct result of a known underlying infection. Concern for neoplasm encompassed any lesion that was suspicious for cancer regardless of biopsy results.

These criteria were analyzed twice by authors and quantitative analysis was performed on the data. University of Alabama at Birmingham Institutional Review Board issued approval IRB-300008415-002.

## Results

There was a total of 37 patient encounters in the year 2019, nine in 2020, and 15 in 2021. Of those in 2020, only one patient presented after March 15, 2020, the beginning of the nationwide shutdown caused by the COVID-19 pandemic [7]. Of the 61 patient encounters at the clinic, 15 (24.6%) were recorded on an electronic health record (EHR), while 46 (75.4%) were documented on paper charts. Documented encounters indicate that 36 (63.2%) of the patients were presenting to the clinic for the first time, and only two (3.5%) patients were insured. Additionally, the median distance each patient had to travel to reach the clinic site was 6.1 miles, with a range of 0 to 56 miles.

**Figure 1:**
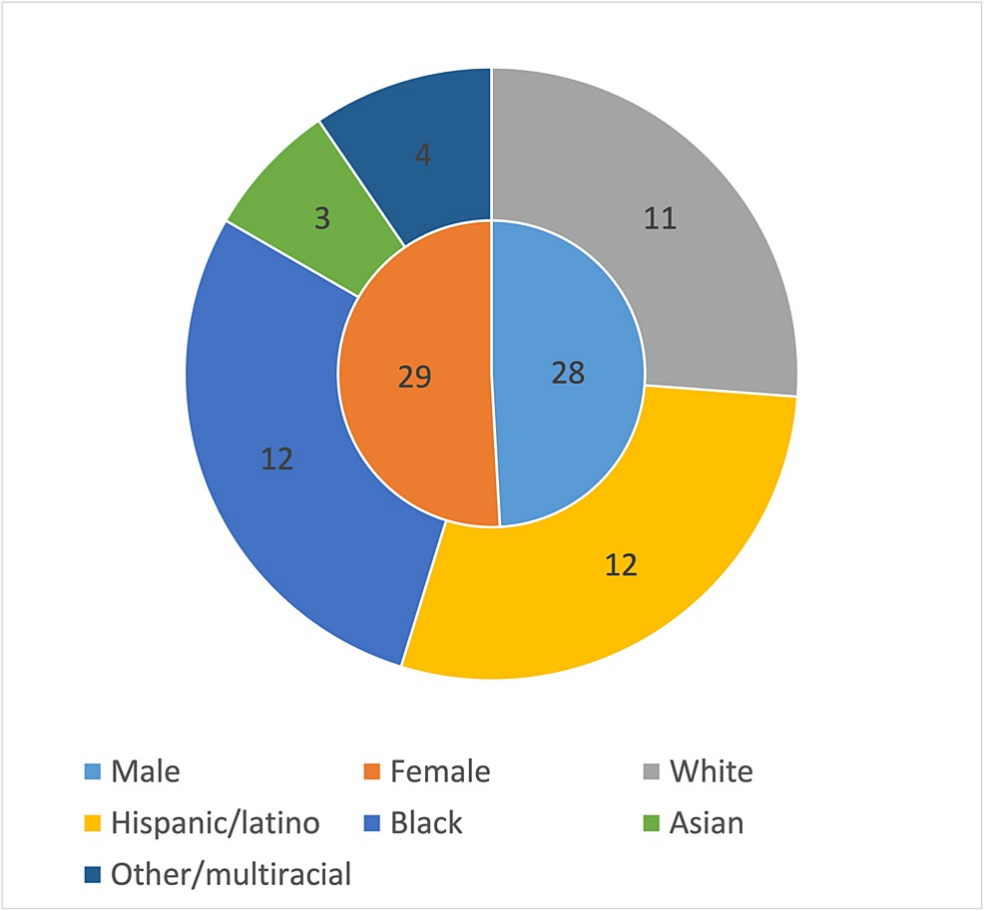
Demographics of patients who presented with a dermatology complaint. There was a total of 28 male and 29 female patients, with a majority of the patients with recorded demographics being Hispanic/Latino or Black. Fifteen patients had no documented ethnicity.

The patient demographics (Figure 1) consisted of 29 (50.9%) females and 28 males (49.1%) with a median age of 40. Of the 42 patients with recorded ethnicities, 12 (28.6%) were Hispanic/Latino, 12 (28.6%) were black, 11 (26.2%) were white, three (7.1%) were Asian, and four (9.5%) were multiracial or of another ethnicity. Of the 33 patients with recorded marital status, 16 (48.5%) were single, 11 (33.3%) were married, and six (18.2%) were divorced. Of the 23 patients with documented education level, 11 (47.8%) had a college degree, eight (34.8%) had a high school diploma/GED, and four (17.4%) had less than a high school education.

**Figure 2:**
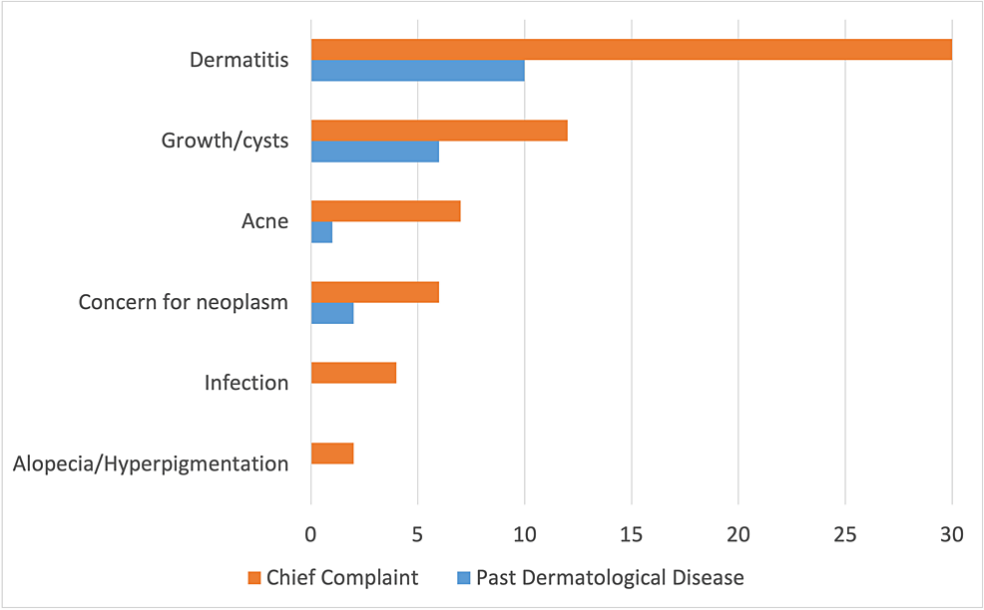
Chief Complaint and Past Dermatological Disease. A total of 18 patients encounters out of 61 had a recorded history of past dermatological diseases.

Out of the 61 patient encounters, 30 (49.2%) presented with chief complaints of dermatitis, 12 (19.7%) with growths/cysts, seven (11.5%) with acne, six (9.8%) with concern for neoplasm, four (6.6%) with concern for infection, and two (3.3%) with alopecia and hyperpigmentation (Figure 2). Only 18 (17.5%) of these patients reported one or more past dermatological disease. Of these 18, 10 (55.6%) patients reported previous dermatitis, six (33.3%) reported previous growths/cysts, two (11.1%) reported previous concern for neoplasm, and one (5.6%) reported previous acne.

Of the 57 patients, 22 (38.6%) reported previous hospitalizations and 26 (45.6%) reported previous surgery, with only six (10.5%) indicating previous dermatological procedures. Other pertinent histories including psychiatric conditions and smoking status were also assessed, with 11 (19.3%) indicating psychiatric comorbidities and 24 (42.1%) indicating significant smoking history (21 current, three former). Fifteen (26%) patients also reported medication allergies.

**Figure 3:**
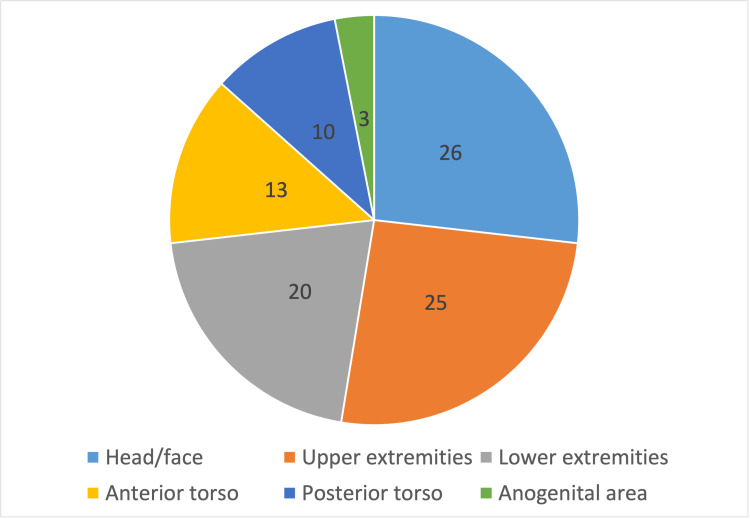
Location of Dermatology Condition on the Body. Location of skin condition was recorded in a total of 58 patient encounters. In 15 cases, the condition was in two separate locations. In nine cases, the condition was in three or more locations.

Location on the body of each dermatological complaint was assessed (Figure 3). Reported locations include 26 on the head/face, 25 on the upper extremities, 13 on the anterior torso, 10 on the posterior torso, 20 on the lower extremities, and three located in the anogenital area. Pathology was localized to a single location in 34 patients, two locations in 15 patients, three in six patients, and three or greater in three patients.

**Figure 4:**
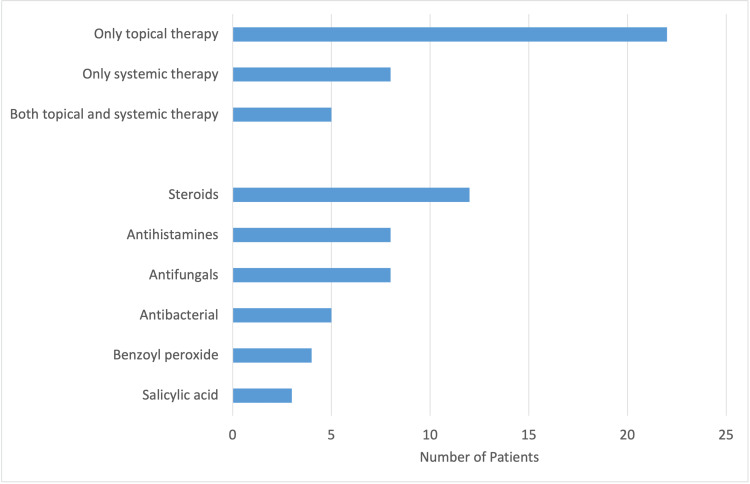
Treatments Used at the Free Health Clinic for Dermatologic Conditions. A total of 40 patients out of 57 underwent treatment with medication only. Seven out of 42 patients received procedural treatment, which included injections, incisions, or biopsies. Two patients received both medication and procedural treatment.

A total of 42 (73.7%) patients that underwent treatment (Figure 4). Thirty-five (83.3%) of these patients received medication, with 22 (52.4%) receiving only topical therapy, eight (19.0%) receiving only systemic therapy, and five (11.9%) receiving both topical and systemic therapy. Twelve (28.6%) received steroids, such as hydrocortisone, clobetasol, or triamcinolone. Eight (19.0%) received antihistamines, such as diphenhydramine, hydroxyzine, or loratadine. Eight (19.0%) received antifungals, such as clotrimazole or fluconazole. Five (11.9%) received antibacterial agents, such as doxycycline or trimethoprim-sulfamethoxazole (TMP-SMX). Four (9.5%) received benzoyl peroxide. Three (7.1%) received salicylic acid. There was a total of seven patients (12.3%) that did not receive treatment due to expense (one patient), lack of biologic agents (one patient), lack of procedural availability (three patients), and/or other reasons (two patients). Of the 42 patients that underwent treatment, seven (16.7%) received procedural intervention, such as steroid injections (two patients), punch biopsies (two patients), excisional biopsies (one patient), and lancing and drainage (one patient).

**Figure 5:**
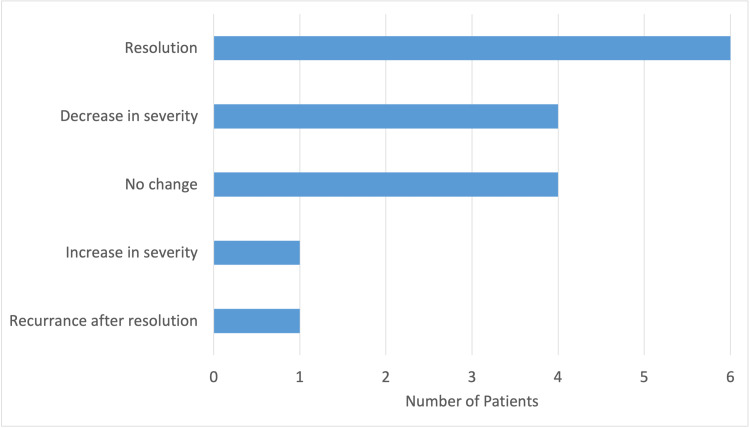
Effectiveness of Treatment. A total of 16 patients out of 57 were able to follow up.

Twenty-one (36.8%) patients were asked to follow up at the clinic with a median and mode follow-up period of 30 days. The longest follow-up period was one year. A total of 16 (76.2%) patients followed up, with only 12 (57.1%) of those within the recommended time frame. In the cohort that followed up, the dermatological condition resolved in six (37.5%) patients, decreased in severity in four patients (25%), did not change in four patients (25%), increased in severity in one patient (6.3%), and recurred after resolution in one patient (6.3%) (Figure 5). Eleven (19.3%) patients required a referral to charity care (two patients), a dermatology clinic (eight patients), or general surgery (one patient). Of those that followed up, only three (18.8%) patients had complications with medication adherence. One patient required immediate hospitalization, but it is unclear if this was secondary to their dermatological condition or a different underlying condition.

## Discussion

There are many barriers to adequate treatment for patients utilizing free health clinics for dermatological needs [8]. In populations within the Southern United States, some of these barriers may be even more difficult to overcome. For example, patients may be unable to minimize sun exposure due to geographic location or the nature of their work or labor [9]. For those who spend a greater amount of time outdoors, due to work or homelessness, it may be difficult to maintain adequate hygiene, and, as a result, they may struggle to keep wounds clean or be unable to properly care for necessary post-procedural wounds, increasing risk of infection or other complications [10]. These patients are also more likely to have difficulty traveling to the clinic site, as they may not have a personal vehicle and often heavily rely on public transportation or walkability [10]. Additionally, due to the majority of patients being uninsured, as seen in this study with 96.5% of presenting patients being uninsured, it may be difficult for them to afford necessary treatment [11].

In this study, 71 patients with dermatological complaints were seen from 2019-2021. This number of patients is likely lower than normal due to the nationwide shutdowns seen beginning in early 2020 secondary to the COVID-19 pandemic. The prevalence of dermatological complaints, therefore, were inconsistent with previous years [12,13]. Additionally, it is unclear if the prevalence of dermatology complaints in our study is comparable to other free health clinics during this time due to there being limited studies on this topic. Despite having 71 patients present with dermatological complaints for this study, only 57 were able to be used due to insufficient recorded data regardless of whether patient information was recorded in an EHR or paper chart. Unfortunately, other studies have also shown this issue of accurate record keeping being an issue in free health clinics [14].

The most common ethnicities for patients in our study included Black (28.6%) and Hispanic/Latino (28.6%). Black patients were underrepresented in our study as the metropolitan area the clinic serves is 68.3% black [15]. Conversely, Hispanic/Latino patients were overrepresented as they account for only 4.1% of the metropolitan area [15]. These demographics likely differ from other regions of the United States. For example, a study conducted in an urban free clinic in Milwaukee, Wisconsin, had a majority of respondents identifying as Black (60%) [11]. These findings echo another issue in educating not only dermatologists but also primary care physicians about disease representation in skin of color. Dermatology conditions in skin color are often not as established in textbooks and not often represented in other educational materials [16]. As skin of color comprises the majority of dermatology patients in this study, it is exceedingly important to provide adequate education to patients, medical students, and physicians regarding dermatological complaints within this group.

**Figure 6:**
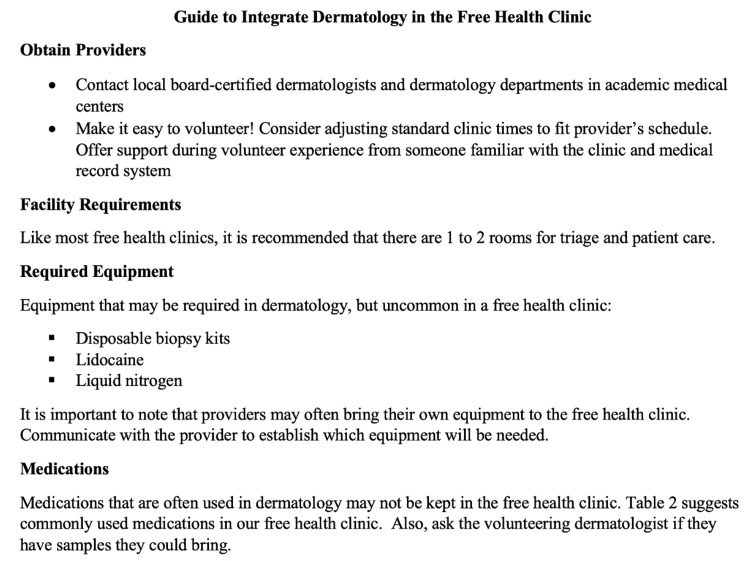
Recommendations for Establishing a Dermatology Clinic in the Free Healthcare Setting based on the Experience of the Studied Clinic

The most common complaints made within the study population were dermatitis, growths/cysts, and acne (Figure 2). In comparison to the general population, particularly the elderly, chief complaints are generally eczematous dermatitis (21.7%), pruritus (19.6%), and fungal infections (16.7%) [17]. It is difficult to establish a correlation between these two groups as our sample size was small. The more commonly used therapies in this clinic included topical and systemic steroids, antihistamines, and antifungals, all of which are commonly used in private clinics [18]. Biological therapy, electrodessication, Mohs surgery, and laser therapy, among other interventions, were not available at this free health clinic. Differences seen in treatment options are likely due to treatment cost and specialist availability. A summary of equipment and medications required to operate a dermatology clinic based on the clinic in this study can be found in the appendix (Figure 6, Table 2).

It is important to note that the COVID-19 pandemic and its resulting nationwide shutdown may have led to skewed data, as the number of clinic days was drastically decreased. Early in the pandemic, the number of visits to ambulatory care practices declined by nearly 60%, decreasing patient follow-up [19]. In the future, telemedicine may be a fruitful method to address these issues and increase patient accessibility to health care within dermatology. Telemedicine allows patients to see their providers without having to make travel arrangements that are often exceedingly difficult for this patient population to obtain [20]. With this in mind, increasing free or public access telemedicine capability platforms may be helpful for this population.

There are several limitations of this study. The documentation used for this study was suboptimal, with several paper charts providing little to no information regarding the presenting complaint, provided treatments, and outcomes. The available patient records also only dated as far as 2019 due to limited storage within the clinic, so confounding variables introduced by the COVID-19 pandemic were unable to be delineated. Although this study represented all recorded patients seen at the free health clinic, it was limited to just one free health clinic. Other free health clinics in the same region were surveyed for study inclusion but were ultimately rejected as they did not have well-documented dermatological findings or did not host dermatology specialty clinics. Additionally, this study was limited to an urban city in the Southern US, so data may not be able to be extrapolated to other populations.

Future directions include looking at a broader range of free health clinics, in both urban and rural areas across the nation, increasing the quality of recorded information in either EHR or paper charts, and providing physicians, including dermatologist and non-dermatologist physicians alike, with a standardized note for dermatology encounters. With a large enough sample size, comparative analysis can be done within subgroups of the data to improve study inferences.

## Conclusions

To our knowledge, this is the first study to analyze dermatology complaints and the capacity to treat them in the free health care setting. With the high prevalence of dermatology complaints in the underserved population, it is imperative to understand potential barriers to care, treatment options, and available external resources for these patients.

## Appendices

**Table 2:** Medications Commonly Used in the Studied Clinic for Dermatological Complaints

Drug Class	Topical	Oral
Steroids	Hydrocortisone 1-2.5% cream or ointment, Triamcinolone cream or ointment, Clobetasol cream or ointment	Prednisone
Antifungals	Clotrimazole cream, Ketoconazole 2% shampoo	Fluconazole
Antibiotics	Mupirocin	TMP/SMX (Bactrim), Doxycycline
Acne/Folliculitis treatment	Clindamycin solution, Benzoyl Peroxide wash, Adapalene (Differen) gel	
Antihistamines		Hydroxyzine, Diphenhydramine (Benadryl), Loratadine (Claritin)
